# An observational study of individual child journeys through autism diagnostic pathways, and associated costs, in the UK National Health Service

**DOI:** 10.3389/fresc.2023.1119288

**Published:** 2023-05-25

**Authors:** Ian Male, William Farr, Stephen Bremner, Heather Gage, Peter Williams, Emma Gowling, Emma Honey, Aaron Gain, Jeremy Parr

**Affiliations:** ^1^ Research Department, Sussex Community NHS Foundation Trust, West Sussex, United Kingdom; ^2^ Primary Care and Public Health, Brighton and Sussex Medical School, Brighton, United Kingdom; ^3^ Faculty of Education, University of Cambridge, Cambridge, United Kingdom; ^4^ School of Biosciences and Medicine, University of Surrey, Guildford, United Kingdom; ^5^ School of Mathematics, University of Surrey, Guildford, United Kingdom; ^6^ Geriatrics, Queen Alexandra Hospital, Cosham, United Kingdom; ^7^ Paediatrics, Northumberland Tyne and Wear National Health Service, Newcastle Upon Tyne, United Kingdom; ^8^ Public Health, University of Brighton, Brighton, UK; ^9^ Population Health Sciences Institute, Newcastle University, Newcastle Upon Tyne, United Kingdom; ^10^ Faculty of Medical Sciences, Great North Children's Hospital, Newcastle upon Tyne NHS Foundation Trust, Newcastle Upon Tyne, United Kingdom

**Keywords:** autism, diagnostic process, health economics, cost of assessment, diagnostic pathways, paediatrics, child

## Abstract

**Background:**

Demand for diagnostic assessment in children with possible autism has recently increased significantly. Services are under pressure to deliver timely and high-quality diagnosis, following National Institute and Care Excellence multidisciplinary assessment guidelines. This UK National Health Service study aimed to answer: how many hours of health professional time are required to deliver autism diagnostic assessment, and how much does this cost?.

**Method:**

Case notes of 20 children (1–16 yrs.) from 27 NHS trusts, assessed through an autism diagnostic pathway in the previous year, were examined retrospectively. Data included: hours of professional time, diagnostic outcome. Assessment costs calculated using standardised NHS tariffs.

**Results:**

488 children (aged 21–195 months, mean 82.9 months, SD 39.36) from 22 Child Development Services (CDS), four Child and Adolescent Mental Health Services (CAMHS) and one tertiary centre; 87% were either under 5 (36%) or 5 to 11 years (51%). Children seen by CDS were younger than CAMHS (mean (SD) 6.10 (2.72) vs. 10.39 (2.97) years, *p* < 0.001). Mean days to diagnosis were 375 (SD 235), with large variation (range 41–1553 days). Mean hours of professional time per child was 11.50 (SD 7.03) and varied substantially between services and individuals. Mean cost of assessment was £846.00 (SD 536.31). 339 (70.0%) children received autism diagnosis with or without comorbidity; 54 (11%) received no neurodevelopmental diagnosis; 91 (19%) received alternative neurodevelopmental diagnoses. Children with one or more coexisting conditions took longer to diagnose, and assessment was more costly, on average 117 days longer, costing £180 more than a child with no neurodevelopmental diagnosis. Age did not predict days to diagnosis or assessment costs.

**Conclusion:**

Typical assessment took 11 h of professional time and over 12-months to complete, costing GB£850 per child. Variation between centres and children reflect differences in practice and complexity of diagnostic presentation. These results give information to those delivering/planning autism assessments using multi-disciplinary team approach, in publicly funded health systems. Planning of future diagnostic services needs to consider growing demand, the need for streamlining, enabling context appropriate services, and child/family complexity.

## Introduction

In the UK National Health Service (NHS), the National Institute for Health and Care Excellence (NICE) Autism Diagnostic Guidelines (1) recommend a multidisciplinary approach to the diagnostic assessment of children with Autism Spectrum Disorder (Autism). This assessment should also consider differential diagnosis, and in the context of an autism diagnosis, identify co-occurring mental health and neurodevelopmental conditions (1, 2). In children, it is recommended that the core diagnostic team should include an experienced paediatrician or child psychiatrist, working alongside a clinical, or educational, psychologist, and a speech and language therapist (SALT), to build a picture of the child across settings (1). The team may sit within Child Development/Paediatric services (CDS), Child and Adolescent Mental Health Services or Children and Young People's Services (CAMHS), or Tertiary settings.

Recent increases in waiting times for diagnosis have led to parental dissatisfaction (3–5), prompting the NHS England Long Term Plan (6) to seek solutions, including innovative models of service delivery to help address waiting times. Although various challenges have contributed to long waiting times, including workforce availability, increased case complexity and a funding model that is not responsiveness to demand (7), the greatest difficulties have related to increased demand for diagnostic assessment, which has doubled in the UK from 2015 to 19 (8). This is in line with international prevalence studies (9), with recent studies suggesting prevalence in children may be as high as 1 in 50 (10, 11) or even 1 in 30 (12, 13). Most UK services are resourced on, at best, a prevalence of 1 in 100 (14).

The process of autism diagnosis is lengthy, and smaller study by this research team (15), based on descriptions from lead clinicians across 12 UK centres of their standard autism diagnostic pathway, found that the typical journey of a child through that pathway in 2013 took 13 h of health professional time to complete, costing £800 (US$1200) per child. However, this study did not look at individual child journeys and how much these varied from the submitted standard pathway. Whilst approaches to diagnostic assessment and funding healthcare vary internationally, this gave a helpful starting point in understanding the resources required to deliver a multidisciplinary diagnostic assessment for possible autism (15). It also provided evidence for the NHSE Transforming Care Program that subsequently fed into the Long-Term Plan for the NHS (6).

These results, however, were based on a “typical” autism assessment and what health professionals reported they do, which may not have necessarily reflected the actual journeys of individual children through each pathway. The current study was therefore designed to collect detailed data retrospectively on the diagnostic journeys of individual children to enable comparisons of the time taken and professionals seen within and between teams in CDS, CAMHS and Tertiary centres.

The study aims were to:

1. Explore, by service and type of service, the number of stages in the assessment process, and the time taken from referral to diagnosis and number of types of professionals involved.
2. Investigate the effect of age of child and final diagnosis on stages, time taken and professional involvement.
3. Estimate the cost of the process (based on professional time input) at the level of individual children and explore cost drivers (age, diagnosis, co-existing conditions).
4. Inform decisions about the appropriate resourcing of centres providing diagnostic services for children with possible autism.

## Method

### Recruitment of services

Clinical teams (in CDS, CAMHS or Tertiary centres) and lead clinical team members such as Consultant Paediatricians, were identified through the British Academy of Childhood Disability (BACD) and British Association of Community Child Health (BACCH), and *via* teams self-identifying through the National Institute for Health Research Portfolio.

### Participants

Children aged 1–16 years who (i) had been referred with concerns expressed about possible social communication difficulties, or possible autism or (ii) had been identified at initial assessment as needing further assessment of social communication, or possible autism, even if the initial referral was for another reason, e.g., “Looked After Child”, or possible Developmental Coordination Disorder or ADHD. The exclusion criteria were: Age over 16 years; no concerns expressed about possible autism; or parental consent not obtained.

### Procedure

Following obtaining parental consent, and where appropriate child assent, data were extracted from the case notes of children who had completed their diagnostic pathway for possible autism within the 12 months from the point at which the service commenced study participation. Data was collected from case notes by a member of staff either from their research or clinical team based at the site. All data collection analysed in this paper was retrospective. Four centres also collected data prospectively to explore feasibility of this approach, including one tertiary centre that only collected data prospectively (data not included in this paper).

Services were asked to complete a standardised, anonymised proforma (see supplemental files) for each child recruited. This included:

– age of child
– information about the child for up to three separate information gathering sessions (at any point in the process) e.g. school questionnaire or observation, and the professionals involved.
– the date and professional involvement at each stage of the assessment process; space was provided for up to four meetings/sessions about each child (filtering for referrals; initial assessment, and referral for further assessment if autism was suspected; two further assessments). A diagnostic conclusion could be reached for some children without all these stages
– final diagnosis (no neurodevelopmental diagnosis, a different neurodevelopmental diagnosis (not autism), autism only, autism with co-occurring conditions)
– referral and follow up of those diagnosed with ASD with date and professionals involved
– Professional involvement was categorised as: Paediatrician; Psychologist; Speech and Language Therapist (SALT); Nurse/Health Visitor/Community Nursery Nurse; Administrator; Other); and the banding (pay grade) and time spent by professionals at each encounter.
– Additional contacts such as phone calls, parental support, child intervention, and completion of Education, Health and Care Plans were not included.

### Sampling

A consecutive opportunistic sample of 20 children completing diagnostic assessment for possible Autism consecutively was sought from each service. The sample in each service was restricted to 20 for pragmatic reasons, i.e., to reduce the burden on staff and encourage participation. With multiple services involved, it was expected that the number of children by type of service (CDS, CAMHS, Tertiary) would be sufficient to enable comparisons in an observational study.

### Analysis

Descriptive statistics by individual service and service type were produced for age of children and final diagnosis, using Stata Version 16. Inferential analysis (described below) was carried out using SPSS Version 28. The assessment process to diagnosis (four possible stages of filtering, initial assessment/referral and two assessments) was described by site and type of service according to the numbers of days, stages and professionals seen. Information gathering was excluded as this occurs at any point in the overall process and the dates of those events were not available. The mean number of children having information visits and follow up visits were calculated by site, type of service and number of professionals seen. The number of children seeing each professional on at least one occasion (excluding during follow up) and the mean number of hours per child were calculated.

Costs were calculated (in British pounds, 2020) at the level of the individual child from the product of professional time (according to type and band) using nationally validated tariffs (16). Overheads for administration and management are included in national tariffs so time reported by some centres for administrators was not included. Mean costs of diagnosis (information gathering and assessment) and total costs (also including follow up) were explored by site and type of service. Bivariate associations were explored between diagnosis costs (includes information gathering) and age of child, final diagnosis and number of days in the four assessment stages to diagnosis using Spearman Rank correlation and Mann-Whitney *U* (MWU) tests. Backward stepwise regression methods were used to model: days in the four assessment stages to diagnosis, with age of child and final diagnosis as predictors; and total costs (information gathering and assessment, excluding post diagnosis follow up) with age of child, final diagnosis, and days in the four assessment stages to diagnosis as predictors. Threshold *p*-value for removal was 0.05, model diagnostics included n, R squared and 95% confidence intervals for each fitted parameter.

### Ethics approval and consent

Research Ethics Committee approval was given by the London Riverside Research Ethics Committee on 23rd March 2017 (17/LO/0512), Health Research Agency approval was given on 12th June 2017 (IRAS ID: 214227). Informed and recorded verbal consent was sought by telephone by local NHS Trust Research and Development Teams, following an approach made to parents by the clinical team. Study Information Sheets were sent or given to parents prior to making telephone contact to seek consent to enter their child into the study. Recruitment occurred between 8/10/2017 and 31/12/2019.

## Results

### Description of sample

**Figure 1:**
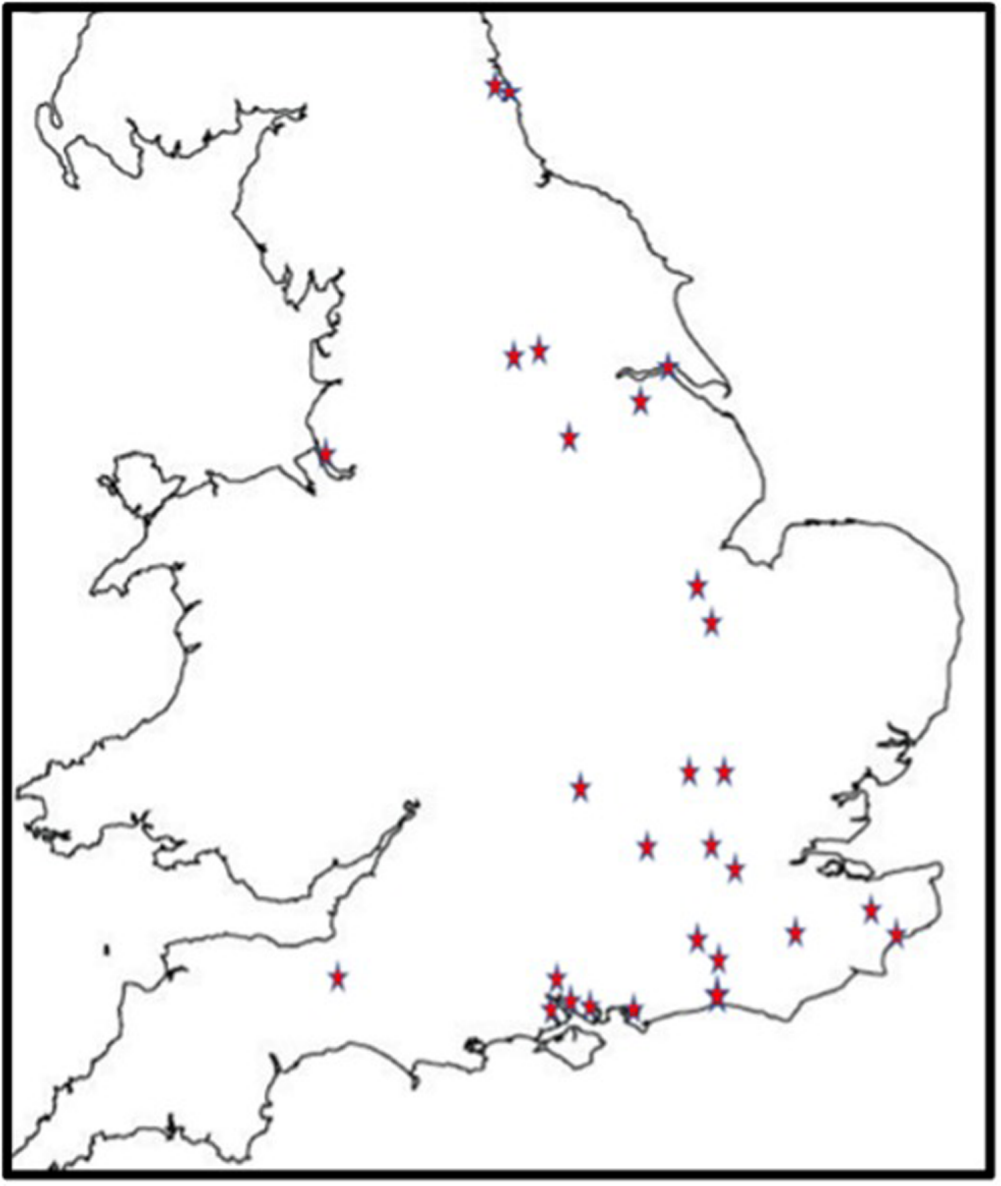
Map showing data collection sites across England.

488 children were recruited from 27 centres (Figure 1). All were based in England. There were 22 CDS recruiting 394 (80.7%) children, four CAMHS (77 children, 15.8%) and one tertiary service (17 children, 4.5%). Final information about diagnosis was available for 484 children, 54 (11.2%) of whom had no autism or other neurodevelopmental condition; 91 (18.8%) were diagnosed with another neurodevelopmental condition, but not autism. The complexity of autism amongst the remaining 339 (70%) children varied as in Table 1, with 148 (30.6%) children identified as having one or more co-occurring neurodevelopmental conditions.

**Table 1:** Final diagnosis of 484 children (no diagnosis was provided for 4 children).

Diagnosis category	*N*	%
1. No ASD	54	11.2
2. No ASD but other condition of importance for neurodevelopmental disorders, e.g. ADHD	91	18.8
3. ASD alone and discharged (level of ASD not serious, and/or support already in place) and no clinic follow up	66	13.6
4. ASD alone—a simple and clear diagnosis, e.g. pre-schoolers,—not discharged and/or follow up needed	125	25.8
5. ASD with singular other neurodevelopmental condition	89	18.4
6. ASD with multiple neurodevelopmental conditions	59	12.2
Total	484	100

**Figure 2:**
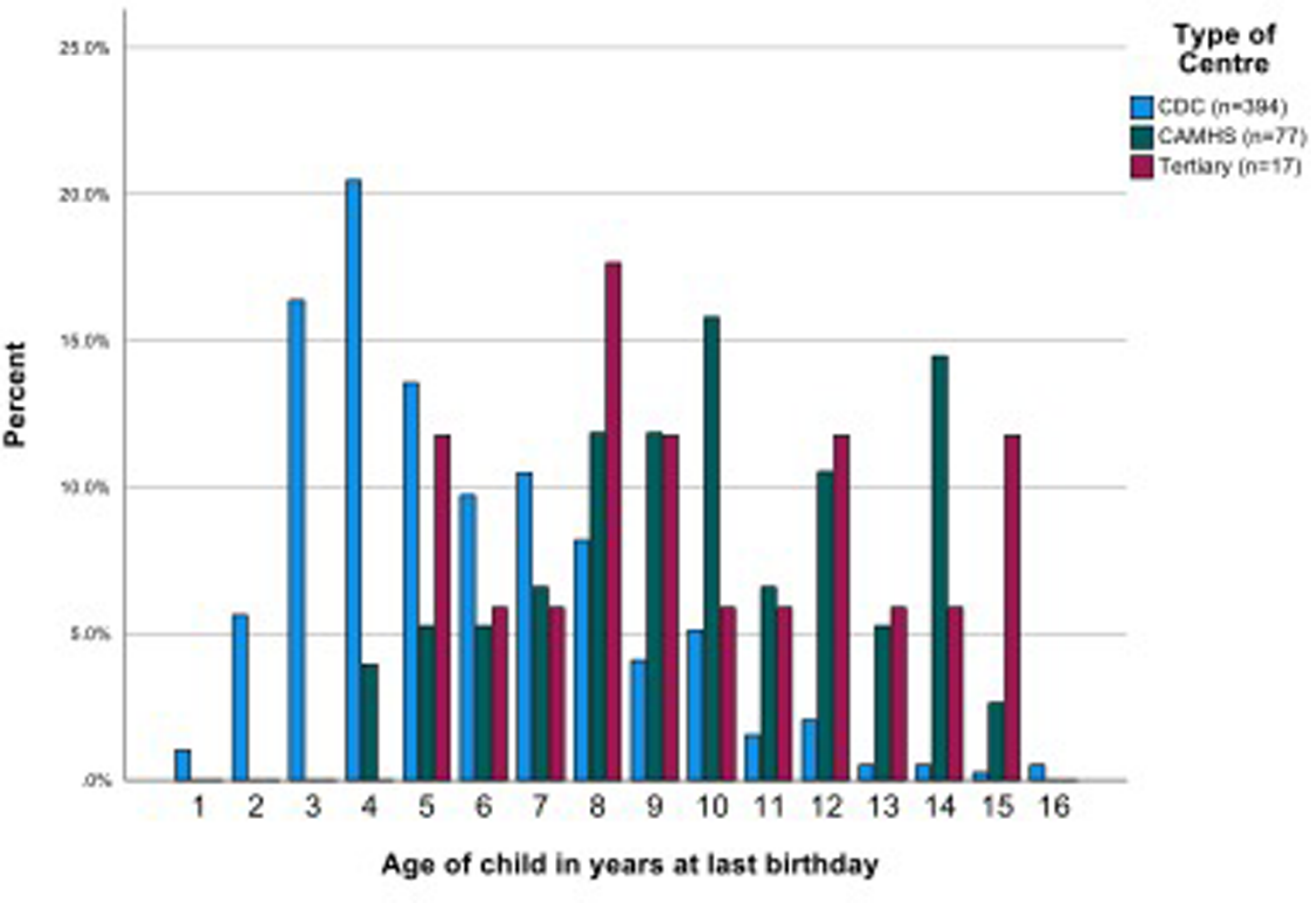
Age distribution of children, by type of centre.

Children's ages ranged from 21 to 195 months (1.75 to 16.3 years) (mean 82.9 months, or 6.9 years, median 72.5, SD 39.36,). The CAMHS team samples included mostly (or only) school aged children. The ages of children seen in CDS varied—four CDS saw pre-school children only, seven CDS provided data on mostly or only school age children and the samples of children from the remaining 11 CDS were mixed pre-school and school aged. Most school age children were of primary school age. Children seen by CDS overall were significantly younger than in CAMHS (mean (SD) 6.10 (2.72) vs. 10.39 (2.97) years, *p* < 0.001). The 17 children referred to the tertiary service were all of school age (mean (SD) 9.82 (3.28) years) which was similar to the children in CAMHS but significantly older than those seen in CDS (Figure 2, Supplementary Material Table S1).

The proportion of children receiving a diagnosis of autism alone (overall 191/484, 39.4%) and autism with one or more co-occurring neurodevelopmental conditions (148/484, 30.6%) varied between centres. Some centres identified most children with autism as having at least one co-occurring conditions (e.g., centre 22: autism alone 1/18, 5.6%; autism with at least one co-occurring neurodevelopmental condition 15/18, 83.3%), whilst others did not record co-occurring conditions in any children (e.g., centre 7: autism alone 14/20, 70%; autism with at least one co-occurring condition 0/20, (0%) (Supplementary Material Table S1).

### Number of stages in assessment, professional involvement and time to diagnosis

The mean length of time across the four assessment stages to diagnosis (filtering, initial assessment with potential referral into formal diagnostic assessment, two further assessments) was just over one-year (mean (SD) 375 (235) days, range 41 to 1553 days) but there was considerable variability both within and between services (Supplementary Material Table S2). The diagnostic process involved all four separate stages for almost one half (47.1%) of children. There were 40 children (8.2%) who did not proceed to further diagnostic assessment following initial clinical contact, of whom 15 were diagnosed with autism, 15 an alternative neurodevelopmental diagnosis, and 10 where autism was ruled out. Most children (74.2%) had at least one information gathering session (overall mean 1.51, SD 1.17). Follow-up was required for 256 children (52.5%), all receiving a diagnosis of autism, whether in isolation or in association with co-occurring condition/s (Table 2).

**Table 2:** Number of professional encounters; 488 children in 27 services.

		Number of professionals seen											
		0	1	2	3	4	5	6	7	8	9	10	Total (%)
Information gathering, *n*, (%)		126 (25.8)	189 (38.7)	124 (25.4)	41 (8.4)	8 (1.6)	0	0	0	0	0	0	488 (100)
Four stages/visits for assessment	1	0	10	26	3	1	0	0	0	0	0	0	40 (8.2)
	2	0	0	42	19	23	6	6	0	0	0	0	96 (19.7)
	3	0	0	0	26	24	34	30	6	1	1	0	122 (25.0)
	4	0	0	0	0	37	32	56	59	25	20	1	230 (47.1)
Total, (%)		0	10 (2.1)	68 (13.9)	48 (9.8)	85 (17.4)	72 (14.8)	92 (18.9)	65 (13.3)	26 (5.3)	21 (4.3)	1 (0.0)	488 (100)
Follow up, *n* (%)		232 (47.5)	217 (44.5)	35 (7.2)	4 (0.8)	0	0	0	0	0	0	0	488 (100)

Number of professional encounters scored by adding the exposure to paediatrician, psychologist, SALT and nurse/HV/CNN at each of the four stages: (filtering, referral, two further assessments, maximum possible = 16.

**Figure 3:**
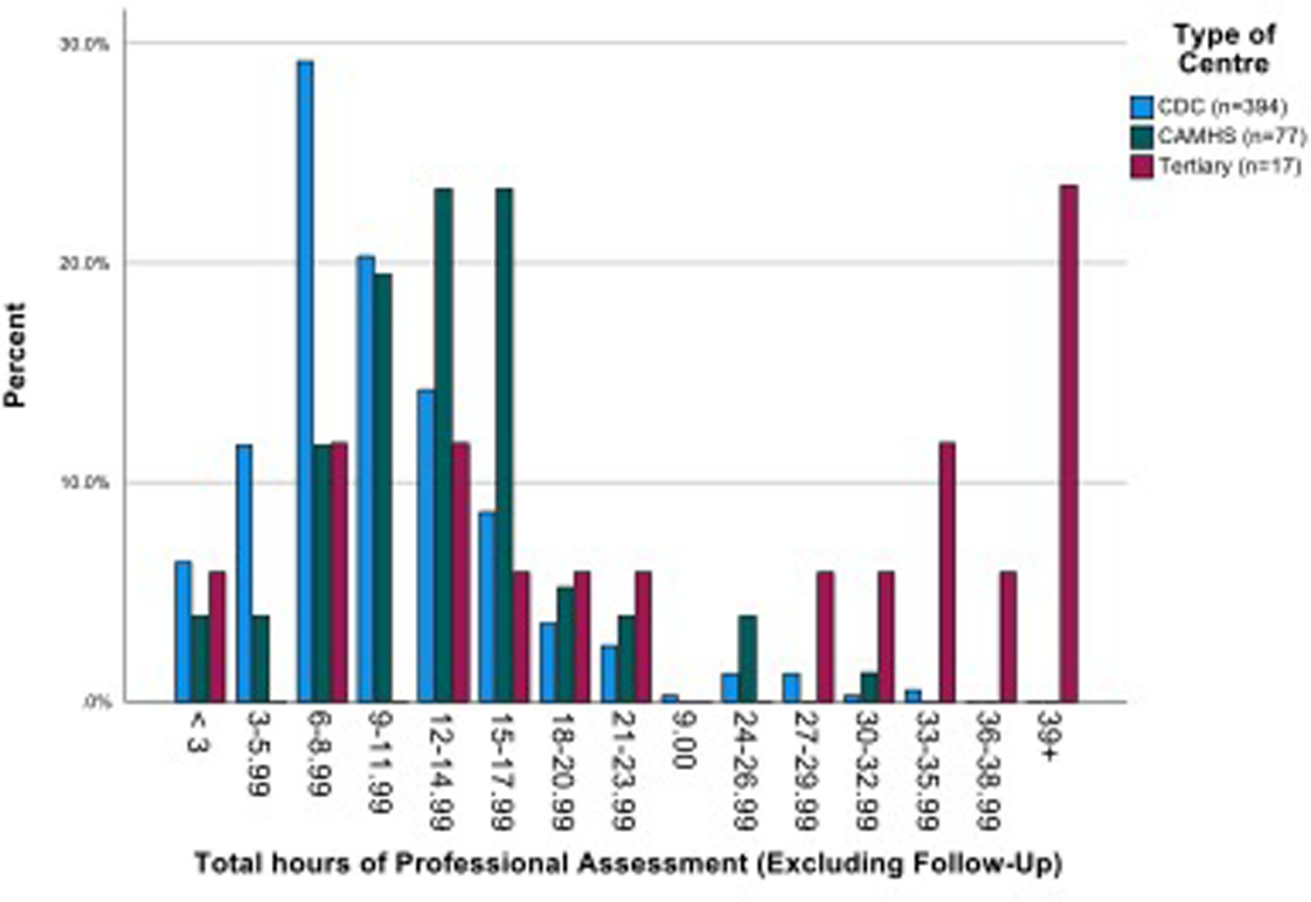
Total hours of professional assessment, excluding follow up, by type of centre.

The mean number of professional contacts across the four assessment stages for each child (sum of involvement of paediatrician, psychologist, SALT and nurse/Health Visitor (HV)/Community Nursery Nurse (CNN)) was 4.27, median 5, maximum 10. The number of professional encounters rose with the number of stages required (Table 2). The diagnosis was based on assessment by two professionals over one or two stages in one fifth of children, and these children were found not to have autism. Paediatricians were involved in the assessments of 434/488 (88.9%) children for a mean (SD) of 3.91 (2.79) hours/child. SALTs were involved in assessing around three quarters of children, and nurses and psychologists saw around one half of the children for lower mean amounts of time. The mean total professional time spent per child was 11.50 (7.03) hours (Table 3). This varied by type of service, being significantly higher in CAMHS than in CDS (mean (SD) 13.26 (5.58) vs. 10.50 (5.75), *p* < 0.001), and as expected, significantly higher in the tertiary centre 26.76 (15.99) than in either of the other two service types (*p* < 0.001 for both comparisons) (Figure 3).

**Table 3:** Professional hours per child.

	*N*		Mean	Median	Std. Dev.	Min.	Max.	Percentiles	
	Valid	Missing						25	75
Paediatrician	488	0	3.91	3.67	2.79	.00	19.25	2.17	5.17
Psychologist	488	0	2.37	.25	3.86	.00	30.25	.00	3.50
SALT	488	0	3.02	2.21	3.49	.00	21.73	.00	4.50
Nurses/Health Visitors	488	0	1.47	.08	2.75	.00	16.17	.00	1.65
All Others (excluding Admin.)	488	0	.73	.00	2.06	.00	26.20	.00	.50
Total Professional	488	0	11.50	10.08	7.03	.00	60.90	7.10	14.40

### Costs

**Figure 4:**
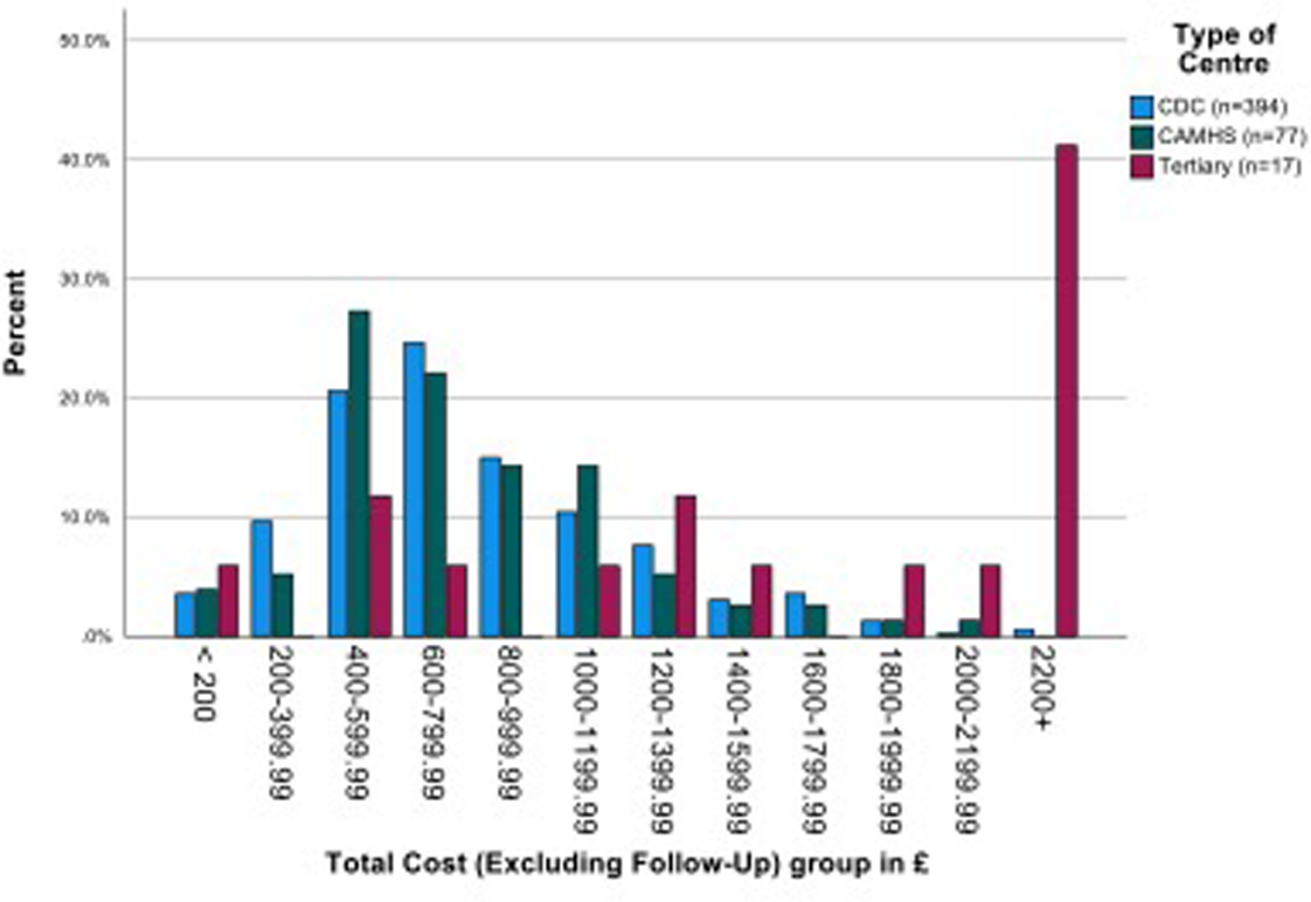
Mean costs per child, (£, 2020), by type of service.

The overall mean (SD) costs of assessment (across four stages, filtering to diagnosis) plus information gathering was £846.00 (s.d. £536.31). There was no significant difference between the mean costs of CDS and CAMHS, but in line with additional staff time required, the mean cost of the tertiary centre assessments was more than double that of both the other services (*p* < 0.001 in both comparisons). When follow up costs are included, the mean costs increased by around £60 to £100. (Figure 4 and Supplementary Material Table S2).

Tests of bivariate associations (Spearman's rho) revealed no association between costs to diagnosis and age (*p* = 0.623), but as expected, there was a highly significant positive association between costs and number of days to diagnosis (*p* < 0.0005). Regression modelling used the log of dependent variables to remove skew in the data. The presence of multiple other neurodevelopmental conditions at final diagnosis alongside autism (diagnosis 6) had significant independent positive association with days to diagnosis; child age was not significant. Having a diagnosis of autism and multiple co-occurring conditions compared to the other diagnoses added 117 days to assessment time. Significant positive associations were found between total costs (excluding follow up) and assessment days to diagnosis, receiving final diagnoses of autism alone but not discharged (diagnosis 4), receiving final diagnosis of autism and one other condition (diagnosis 5) and receiving final diagnosis of autism with multiple conditions. (For modelling details, see Supplementary Material Table S3).

## Discussion

### Summary of main findings

Set in the UK National Health Service, this is the first study to gather data across multiple services on the actual journeys of a large number of children on an autism diagnosis pathway. Overall, 70% of the children received a diagnosis of autism with or without comorbidity, which is in line with the findings from our separate survey across the United Kingdom (8). The study identified long waiting times to completion of diagnosis, of a year or longer, in most centres. This reflects the concerns raised by families, charities and professionals that resulted in the need expressed in the NHS Long-Term Plan (6) for research to find service delivery models to address the current challenges of increasing prevalence and demand for diagnostic assessment. These issues are international, with research on improving the timeliness and quality of diagnosis, and/or the development of new guidelines, occurring in many countries including Australia (17), New Zealand (18), Canada (19–21) and the US (22, 23), as well as in the UK (24–27).

The mean professional time involved in achieving a diagnosis was 11.5 h; the associated mean cost (British pounds, 2020) of £846 per child is similar to an earlier study (conducted in 2013) which looked at standard pathways but did not identify individual child journeys at participating centres (15). This study provides clear evidence of costs between service standards and child journeys. Further, by looking at individual child journeys through the diagnostic pathway, we have identified substantial differences across individual child journeys, whether within or between services; some are discharged following initial assessment, whilst others underwent multiple assessments involving several multi-disciplinary professionals. As expected, those attending a tertiary centre required more detailed assessment, reflecting diagnostic complexity of children referred for tertiary assessment, explaining a doubling in cost. The presence of one or more comorbidities was associated with a longer overall assessment time and higher costs. This is consistent with other studies that have found comorbidity to significantly impact on diagnostic complexity (27). Costs are dependent on the hours of professional time involved and the types of professionals seen, with medical input being the most costly.

### Strengths and limitations of the study

The study addresses an issue of significant patient, public and policy concern. Access to diagnostic services was ranked highly in local and national research priority setting exercises, including that of the Autistica James Lind Alliance (28). By looking at individual child journeys through diagnostic pathways, the study has improved understanding of the professional time involved and resulting costs of assessing children for autism, and of the variations that exist between services, and between individual children at a local level. With 27 centres, and a large sample of 488 children involved, the findings are generalisable to practice in England. Data was collected retrospectively, and may therefore underestimate total costs, for example where patient contacts such as phone calls for parent support were not recorded in the patient record. We did explore the feasibility of prospective data collection in four of the participating centres, but it proved difficult for teams with lengthy pathways to continue recording data through the pathway.

Whilst approaches to service delivery and funding differ internationally, the study provides an indicator for multidisciplinary diagnostic assessment as recommended by NICE (1) and other international guidelines (17, 19). A formal sample size/power calculation was not performed, so tests were exploratory and may be subject to type 1 and type 2 errors. Skewness of the dependent variables (days to diagnosis and total costs) rendered modelling challenging and offered little possibility of high R^2^.

The main challenge encountered was variability in the quality of data recording between sites. As each site utilised a member of staff either from their research or clinical team to collect data from case notes, differences may have occurred in their understanding of the pathway, familiarity with recording processes and means of reporting of diagnostic outcomes. Difficulties may also have arisen in determining the different stages of the diagnostic process as the local terminology used to describe assessments varied (e.g., for the first assessment, the terms “triage clinic”, “filter clinic”, “stage one clinic” and “general developmental assessment” were all used). Whilst data were recorded on formal assessments, we did not collect details on informal contact such as telephone calls with parents between clinics, or linked activities such as support and intervention for the child and family, or completing Education, Health, and Care Needs Assessments, all of which would contribute further to the costs of delivering autism health services, and therefore our findings represent the minimum mean cost for this.

This study was not designed to assess the quality of diagnostic assessment offered by the individual teams although it is interesting to note that, in line with one of the NICE Quality Standards (2), some centres were more likely to identify comorbidity alongside an Autism diagnosis than others. Whilst this may just reflect accuracy of data collection, or differences in case mix across centres, it could also indicate a genuine variation in team approach, with some focussed on answering whether the child has autism, yes or no, whilst others are adopting a broader strength and needs based “neurodevelopmental” approach (29, 30), recognising co-existing conditions and differential diagnosis (24, 27, 30).

### Implications for practice, policy and research

Variation in diagnosis times may be due to several factors. Findings confirmed the conclusions of other studies that case complexity impacts on speed of diagnosis, for example the presence of comorbidities such as ADHD or learning difficulties can result in delays in diagnosis (31). Children presenting with a classic picture of “Autism” who are non-verbal and may have had language regression with very obvious repetitive and sensory driven behaviours may, by contrast, be recognised more easily (32), and may therefore be suitable for a more abbreviated, streamlined or tiered diagnostic assessment (22, 30). Effective initial assessment by an experienced diagnostician, may also enable a proportion of children to be recognised as not needing a full Autism diagnostic assessment early in the process, either leading to early discharge or referral to alternative pathways such as community SALT, and avoidance of the costs of the full assessment. Speed of diagnosis may, however, also depend on family factors, including reliability of clinic attendance, and on local funding. Many teams across the UK face challenges in recruiting and retaining staff across the multidisciplinary team (8). Well-resourced teams are under less pressure in providing a timely service than small under resourced teams where morale is low and waiting lists long (33). Equally, it may be easier to operate a timely service where there are good relationships with other agencies such as Early Help, education and social care that can provide detailed high quality assessment, intervention and information gathering prior to specialist referral (22, 30) and allowing more bespoke approaches to individual child assessment.

The current emphasis on identifying novel and more efficient approaches to assessing children for possible Autism, to reduce waiting times and improve quality of assessment and parental experience has focussed on the skill mix of the multidisciplinary team (17, 26, 34, 35). As echoed in our recent survey of practice across 128 UK services (8), some centres have utilised staff on lower pay bandings such as community nursery nurses, early years practitioners and psychology assistants to deliver parts of the pathway (e.g., collating information from questionnaires and in observational assessments in educational settings or clinics using tools such as ADOS and Qb Test (30)). Elsewhere, teams have utilised the skills of nursing staff, including health visitors and psychiatric nurses, or teachers, for example in triage clinics, history taking and post diagnosis support clinics. Whilst it is not possible, to comment on the quality of assessment completed when comparing one professional group with another, it is evident that such approaches are being used in other areas of healthcare (36, 37).

Use of skill mix is a solution to the problem of long waiting times to diagnosis that has been adopted internationally (8). Other approaches have also been suggested that offer potential for the UK National Health Service. These include: tailored assessments for children with “obvious Autism” (17, 34, 35); broadening the workforce through training (38); improving quality of information available at the time of referral (8, 30); improving support for the family throughout the process (39, 40); use of single practitioner assessment (17, 41, 42), although there remain concerns that this may not be as reliable as multidisciplinary assessment (1).

Going forward, a well-resourced and skilled multidisciplinary workforce, including professionals from mental health and paediatric/child development teams, is required to deliver a high quality and timely service to children and families which might be delivered by a broader single Neurodevelopmental Pathway in an integrated CDS/CAMHS service to consider Autism, ADHD and other neurodevelopmental conditions (30, 43). This study provides a baseline to aid commissioners and provider organisations to appropriately resource future diagnostic services, although for those seeking to develop a neurodevelopmental approach including resources for support and intervention, this would require further investigation.

## Data Availability

The original contributions presented in the study are included in the article/**Supplementary Material**, further inquiries can be directed to the corresponding author.
