# Supplementary material for: An observational study of individual child journeys through autism diagnostic pathways, and associated costs, in the UK National Health Service
Source: Front Rehabil Sci. 2023 May 25;4:1119288. doi: 10.3389/fresc.2023.1119288 (PMC10283036; doi:10.3389/fresc.2023.1119288)
Supplement: Supplementary file 1 [file Datasheet1.pdf]

Supplementary Table 1: Ages and final diagnoses of children by service and type of service

Type of service	Site ID	Number of children (% of All)	Number pre-school n (as reported by service)	Number school n (as reported by service)	Mean Overall Age in months (SD)	Pre-school: Mean Age in months (SD)	School: Mean Age in months (SD)	Diagnosis1 None Total n (% within site)	Diagnosis2 No ASD, other condition n (% within site)	Diagnosis3 ASD no follow up required n (% within site)	Diagnosis4 Just ASD follow up required n (% within site)	Diagnosis5 ASD + 1 other condition n (% within site)	Diagnosis6 ASD + >1 other conditions n (% within site)
Tertiary	25	17 (3.5%)	0	17	117.9 (39.4)	n/a (n/a)	117.9 (39.4)	3 (17.6%)	2 (11.8%)	9 (52.9%)	0 (0.0%)	3 (17.6%)	0 (0.0%)
CAMHS – mostly, only school age	4	20 (4.1%)	2	18	109.8 (33.6)	67.0 (0.0)	114.6 (32.0)	5 (25.0%)	1 (5.0%)	0 (0.0%)	13 (65.0%)	1 (5.0%)	0 (0.0%)
	7	20 (4.1%)	0	20	132.8 (27.5)	n/a (n/a)	132.8 (27.5)	4 (20.0%)	2 (10.0%)	3 (15.0%)	11 (55.0%)	0 (0.0%)	0 (0.0%)
	11	17 (3.5%)	3	14	105.2 (35.5)	55.0 (1.0)	115.9 (29.1)	3 (17.6%)	1 (5.9%)	9 (52.9%)	2 (11.8%)	2 (11.8%)	0 (0.0%)
	26	20 (4.1%)	0	20	149.5 (29.4)	n/a (n/a)	149.5 (29.4)	2 (10.0%)	0 (0.0%)	8 (40.0%)	2 (10.0%)	8 (40.0%)	0 (0.0%)
	ALL	77 (15.8%)	5	72	124.7 (35.7)	59.8 (6.6)	129.3 (32.2)	14 (18.2)	4 (5.2)	20 (26.0)	28 (36.4)	11 (14.3)	0 (0.0)
CDS – mostly, only school age	2	20 (4.1%)	0	20	116.8 (33.5)	n/a (n/a)	116.8 (33.5)	3 (15.0%)	6 (30.0%)	3 (15.0%)	6 (30.0%)	2 (10.0%)	0 (0.0%)
	9	19 (3.9%)	1	18	103.5 (40.5)	54.0 (n/a)	106.2 (39.8)	0 (0.0%)	1 (5.3%)	12 (63.2%)	2 (10.5%)	3 (15.8%)	1 (5.3%)
	16	9 (1.8%)	1	8	65.0 (21.1)	28.0 (n/a)	69.6 (17.1)	0 (0.0%)	2 (22.2%)	0 (0.0%)	5 (55.6%)	2 (22.2%)	0 (0.0%)
	20	10 (2.0%)	0	10	89.7 (20.5)	n/a (n/a)	89.7 (20.5)	0 (0.0%)	0 (0.0%)	1 (10.0%)	8 (80.0%)	1 (10.0%)	0 (0.0%)
	23	20 (4.1%)	2	18	72.1 (15.8)	52.0 (5.7)	74.3 (15.0)	2 (10.0%)	0 (0.0%)	1 (5.0%)	3 (15.0%)	3 (15.0%)	11 (55.0%)
	24	14 (2.9%)	1	13	103.9 (31.8)	51.0 (n/a)	107.9 (29.1)	5 (35.7%)	2 (14.3%)	4 (28.6%)	1 (7.1%)	2 (14.3%)	0 (0.0%)
	29	20 (4.1%)	0	20	90.0 (38.4)	n/a (n/a)	90.0 (38.4)	1 (5.0%)	18 (90.0%)	0 (0.0%)	0 (0.0%)	0 (0.0%)	1 (5.0%)
	ALL	112 (23.0%)	5	107	93.6 (35.0)	47.4 (11.3)	95.7 (34.2)	11 (9.8)	29 (25.9)	21 (18.8)	25 (22.3)	13 (11.6)	13 (11.6)

Site Type	Site ID	Number of children (% of All)	Number pre-school n (as reported by service)	Number school n (as reported by service)	Mean Overall Age in months (SD)	Pre-school: Mean Age in months (SD)	School: Mean Age in months (SD)	Diagnosis1 None Total n (% within site)	Diagnosis2 No ASD, other condition n (% within site)	Diagnosis3 ASD no follow up required n (% within site)	Diagnosis4 Just ASD follow up required n (% within site)	Diagnosis5 ASD + 1 other condition n (% within site)	Diagnosis6 ASD + >1 other conditions n (% within site)
CDS – Mixed ages	1	21 (4.3%)	8	13	64.8 (26.4)	37.5 (8.5)	81.6 (17.8)	0 (0.0%)	3 (15.0%)	3 (15.0%)	2 (10.0%)	6 (30.0%)	6 (30.0%)
	5	17 (3.5%)	6	11	69.7 (29.0)	37.5 (6.0)	87.3 (19.1)	0 (0.0%)	1 (5.9%)	0 (0.0%)	3 (17.6%)	9 (52.9%)	4 (23.5%)
	6	15 (3.1%)	5	9	72.5 (28.3)	41.0 (8.9)	92.3 (13.1)	2 (13.3%)	3 (20.0%)	0 (0.0%)	8 (53.3%)	2 (13.3%)	0 (0.0%)
	8	21 (4.3%)	7	14	79.6 (28.9)	47.4 (7.0)	95.6 (20.6)	0 (0.0%)	15 (71.4%)	0 (0.0%)	3 (14.3%)	2 (9.5%)	1 (4.8%)
	10	20 (4.1%)	3	17	92.9 (36.7)	43.3 (5.7)	101.6 (32.4)	1 (5.0%)	0 (0.0%)	0 (0.0%)	6 (30.0%)	7 (35.0%)	6 (30.0%)
	12	19 (3.9%)	12	7	56.1 (30.1)	37.3 (9.0)	88.4 (25.3)	2 (10.5%)	8 (42.1%)	1 (5.3%)	6 (31.6%)	2 (10.5%)	0 (0.0%)
	13	19 (3.9%)	11	8	89.5 (28.8)	69.4 (8.8)	117.1 (22.8)	0 (0.0%)	4 (21.1%)	3 (15.8%)	9 (47.4%)	2 (10.5%)	1 (5.3%)
	14	18 (3.7%)	8	10	70.6 (20.7)	55.8 (17.5)	82.5 (14.8)	13 (81.3%)	0 (0.0%)	1 (6.3%)	2 (12.5%)	0 (0.0%)	0 (0.0%)
	17	13 (2.7%)	4	9	74.5 (22.7)	51.8 (6.6)	84.6 (19.5)	1 (7.7%)	2 (15.4%)	5 (38.5%)	1 (7.7%)	3 (23.1%)	1 (7.7%)
	22	18 (3.7%)	5	13	68.4 (32.1)	33.8 (6.4)	81.7 (27.6)	0 (0.0%)	2 (11.1%)	0 (0.0%)	1 (5.6%)	6 (33.3%)	9 (50.0%)
	27	20 (4.1%)	9	11	58.0 (12.5)	50.2 (8.0)	64.3 (12.1)	2 (10.5%)	0 (0.0%)	2 (10.5%)	8 (42.1%)	3 (15.8%)	4 (21.1%)
	ALL	201 (41.2%)	78	122	72.4 (29.3)	47.1 (14.3)	88.7 (24.6)	21 (10.7)	38 (19.3)	15 (7.6)	49 (24.9)	42 (21.3)	32 (16.2)
CDS – Mostly, only pre-	3	20 (4.1%)	20	0	48.1 (6.6)	48.1 (6.6)	n/a (n/a)	3 (15.0%)	9 (45.0%)	0 (0.0%)	8 (40.0%)	0 (0.0%)	0 (0.0%)
	15	20 (4.1%)	20	0	51.1 (15.0)	51.1 (15.0)	n/a (n/a)	1 (5.0%)	5 (25.0%)	0 (0.0%)	2 (10.0%)	12 (60.0%)	0 (0.0%)

school ages	18	21 (4.3%)	21	0	46.9 (15.3)	46.9 (15.3)	n/a (n/a)	0 (0.0%)	3 (14.3%)	1 (4.8%)	10 (47.6%)	3 (14.3%)	4 (19.0%)
	19	20 (4.1%)	20	0	43.8 (8.0)	43.8 (8.0)	n/a (n/a)	1 (5.0%)	1 (5.0%)	0 (0.0%)	3 (15.0%)	5 (25.0%)	10 (50.0%)
	ALL	81 (16.6%)	81	0	47.4 (12.0)	47.4 (12.0)	n/a	5 (6.2)	18 (22.2)	1 (1.2)	23 (28.4)	20 (24.7)	14 (17.3)
All CDS	22	394 (80.7%)	163	230	73.3 (32.7)	47.4 (13.0)	91.7 (29.8)	37 (9.5)	85 (21.8)	37 (9.5)	97 (24.9)	75 (19.2)	59 (15.1)
**Grand Total**		**27 sites N=488**	**n=169 * 34.5%**	**n=318 * 65.5%**	**82.9 (38.8)**	**47.7 (13.1)**	**101.8 (34.6)**	**n=54 # (11.2%)**	**n=91 # (18.8%)**	**n=66 # (13.6%)**	**n=125# (25.8%)**	**n=89# (18.4%)**	**n=59# (12.2%)**

* The number of PreSchool and School children does not add up to the total number of 488 (missing for 1 child); # The number of final diagnoses does not add up to the total number of 488 (missing for 4 children)

Summary Table

	CDS N=391 Missing 3	CAHMS N=76 Missing 1	Tertiary N=17 Missing 0	Mann Whitney U test
Mean	6.10	10.39	9.82	CDS vs CAHMS p<0.001 CDS vs Tertiary p<0.001 CAHMS vs Tertiary p= .483
Median	5.50	10.33	9.00	
Std. Deviation	2.72	2.97	3.28	
Minimum	1.75	4.50	5.00	
Maximum	16.25	15.92	15.00	
Percentiles 25	4.08	8.52	7.50	
Percentiles 75	7.75	12.71	12.50	

Supplementary Table 2: Number of stages (visits for assessment), professional encounters and time to diagnosis by service and type of service

Site Type	Site ID	Number of children	Four possible assessment stages to diagnosis (filtering, referral, +2)				Cost, includes information gathering		Mean (SD) total cost including follow up	Number of stages (visits) in assessment process					Mean (SD)	
			Mean (SD) days [Missing]	Min. – Max days	Number of professionals seen #: Mean (SD)	Number of professionals seen #: Min - Max.	Mean (SD)	Min. – Max.		Number of visits: Mean (SD)	1 visit n (%)	2 visits n (%)	3 visits n (%)	4 visits n (%)	Inform -ation gather sessions	Follow Up sessions
Tertiary	25	17 (3.5%)	n/a (n/a) [17]	n/a (n/a)	2.94 (0.97)	1 - 4	2060.62 (1355.01)	0.00 – 4087.05	2060.62 (1355.01)	1.53 (0.51)	8 (47.1%)	9 (52.9%)	0	0	2.47 (1.07)	0 (0.00)
CAMHS – mostly, only school age	4	20 (4.1%)	299.9 (127.8) [1]	42 – 628	4.05 (2.11)	1 - 7	644.19 (454.65)	97.50 – 1883.00	1073.12 (584.71)	2.70 (0.86)	1 (5%)	8 (40%)	7 (35%)	4 (20%)	1.10 (0.64)	0.85 (0.37)
	7	20 (4.1%)	475.1 (284.4) [2]	71 - 1288	3.80 (1.70)	1 - 7	703.12 (315.63)	276.00 – 1717.75	708.52 (334.28)	2.40 (0.68)	1 (5%)	11 (55%)	7 (35%)	1 (5%)	2.30 (0.57)	0.05 (0.22)
	11	17 (3.5%)	97.5 (63.0) [2]	55 – 274	1.76 (0.56)	1 - 3	778.50 (295.69)	427.50 – 1446.00	798.88 (290.14)	1.71 (0.47)	5 (29.4%)	12 (70.6%)	0	0	2.88 (0.70)	0.24 (0.44)
	26	20 (4.1%)	383.9 (209.8) [1]	157 - 757	5.85 (1.23)	3 - 8	1035.60 (327.62)	569.00 – 2024.00	1044.40 (325.82)	3.45 (0.69)	0	2 (10%)	7 (35%)	11 (55%)	1.80 (0.83)	0.10 (0.31)
	ALL	77 (15.8%)	324.06 (232.28) [6]	42 - 1288	3.95 (2.08)	1 - 8	790.81 (381.19)	97.50 – 2024.00	910.41 (427.63)	2.60 (0.92)	7 (9.1%)	33 (42.9%)	21 (27.3%)	16 (20.8%)	1.99 (0.94)	0.31 (0.47)
CDS – mostly, only school age	2	20 (4.1%)	423.8 (212.8) [0]	61 – 913	3.75 (0.91)	3 - 6	1117.60 (945.76)	180.00 – 3366.00	1157.65 (970.23)	3.55 (0.51)	0	0	9 (45%)	11 (55%)	2.35 (1.27)	0.50 (0.51)
	9	19 (3.9%)	509.1 (219.5) [0]	59 – 816	2.53 (0.70)	2 - 4	534.37 (152.91)	276.08 – 885.00	542.90 (149.51)	1.79 (0.71)	7 (36.8%)	9 (47.4%)	3 (15.8%)	0	1.42 (0.51)	0.16 (0.37)
	16	9 (1.8%)	205.9 (142.2) [0]	41 – 547	5.67 (0.50)	5 - 6	478.56 (154.22)	222.08 – 777.33	534.39 (142.63)	2.11 (0.33)	0	8 (88.9%)	1 (11.1%)	0	0.89 (0.33)	0.67 (0.50)
	20	10 (2.0%)	240.5 (84.2) [8]	144 - 399	7.20 (1.40)	4 - 9	872.27 (237.63)	507.67 – 1297.67	872.27 (237.63)	3.60 (0.70)	0	1 (10%)	2 (20%)	7 (70%)	1.70 (0.48)	0
	23	20 (4.1%)	382.3 (268.6) [1]	77 – 922	4.20 (1.61)	2 - 7	660.69 (282.70)	198.00 – 1247.50	800.81 (355.61)	3.45 (0.76)	0	3 (15%)	5 (25%)	12 (60%)	1.75 (2.02)	0.80 (0.70)
	24	14 (2.9%)	445.6 (357.4) [0]	103 - 1236	4.07 (1.00)	2 - 6	904.61 (248.79)	606.00 – 1368.00	938.03 (259.53)	2.71 (0.91)	0	8 (57.1%)	2 (14.3%)	4 (28.6%)	1.21 (0.70)	0.36 (0.50)
	29	20 (4.1%)	344.3 (232.1) [0]	77 - 1215	5.95 (1.28)	3 - 8	807.94 (496.05)	295.50 – 1692.40	940.94 (485.13)	3.60 (0.68)	0	2 (10%)	4 (20%)	14 (70%)	0	0.80 (0.41)
	ALL	112 (23%)	388.19 (250.62) [3]	41 - 1236	4.52 (1.79)	2 - 9	781.89 (519.52)	180.00 - 3366.00	847.92 (533.70)	3.03 (0.98)	7 (6.3%)	31 (27.7%)	26 (23.2%)	48 (42.9%)	1.35 (1.29)	0.50 (0.55)

Site Type	Site ID	Number of children	Four possible assessment stages to diagnosis (filtering, referral + 2)				Cost, includes information gathering		Mean (SD) total cost including follow up	Number of stages (visits) in assessment process Mean (SD)						
			Mean (SD) days [Missing]	Min. – Max days	Number of professionals seen #: Mean (SD)	Number of professionals seen #: Min - Max.	Mean (SD)	Min. - Max.		Number of visits: Mean (SD)	1 visit n (%)	2 visits n (%)	3 visits n (%)	4 visits n (%)	Inform -ation gather sessions	Follow Up sessions
CDS – Mixed ages	1	21 (4.3%)	442.4 (125.4) [0]	121 - 623	6.38 (1.43)	4 - 10	855.93 (304.04)	218.17 – 1684.17	891.82 (311.39)	3.38 (0.59)	0	1 (4.8%)	11 (52.4%)	9 (42.9)	1.38 (0.80)	0.38 (0.50
	5	17 (3.5%)	443.4 (193.8) [2]	132 - 781	6.12 (0.93)	4 - 7	822.29 (239.83)	470.17 – 1313.67	944.56 (247.58)	3.24 (0.66)	0	2 (11.8%)	9 (52.9%)	6 (35.3%)	0.59 (0.80)	0.94 (0.56)
	6	15 (3.1%)	489.7 (226.9) [0]	144 - 965	5.73 (1.67)	4 - 8	997.24 (303.59)	573.00 – 1616.50	1065.49 (278.56)	3.60 (0.51)	0	0	6 (40%)	9 (60%)	2.73 (0.80)	0.60 (0.63)
	8	21 (4.3%)	115.2 (78.7) [0]	42 – 422	5.29 (1.15)	2 - 6	615.00 (161.80)	235.00 – 847.50	724.71 (224.72)	3.29 (0.64)	0	2 (9.5%)	11 (52.4%)	8 (38.1%)	1.48 (0.93)	0.67 (0.48)
	10	20 (4.1%)	554.1 (283.0) [0]	185 - 1553	4.80 (0.62)	3 - 6	1481.03 (352.71)	558.00 – 1912.00	1532.32 (364.36)	3.70 (0.57)	0	1 (5%)	4 (20%)	15 (75%)	2.60 (0.60)	0.70 (0.47)
	12	19 (3.9%)	432.0 (234.1) [0]	99 – 824	3.95 (1.22)	2 - 6	565.12 (311.18)	167.33 – 1344.00	607.49 (347.74)	3.32 (0.82)	0	4 (21.1%)	5 (26.3%)	10 (52.6%)	1.21 (0.65)	0.53 (0.51)
	13	19 (3.9%)	344.6 (232.2) [0]	125 - 932	5.16 (1.07)	3 - 6	600.04 (131.91)	378.00 – 908.00	608.56 (133.44)	3.89 (0.31)	0	0	2 (10.5%)	17 (89.5%)	0.05 (0.23)	0.05 (0.23)
	14	18 (3.7%)	536.8 (290.9) [6]	112 - 1119	5.17 (0.79)	4 - 7	388.07 (284.06)	62.35- 1067.50	436.75 (285.57)	2.94 (0.64)	0	4 (22.2%)	11 (61.1%)	3 (16.7%)	0.17 (0.38)	1.0 (0)
	17	13 (2.7%)	345.4 (190.2) [1]	109 - 743	4.77 (1.54)	3 - 8	388.23 (147.55)	216.00 – 621.00	425.61 (140.18)	3.62 (0.51)	0	0	5 (38.5%)	8 (61.5%)	0.23 (0.44)	0.46 (0.52)
	22	18 (3.7%)	343.3 (115.9) [0]	144 - 599	7.33 (0.69)	6 - 9	1138.05 (233.50)	666.33 – 1626.83	1227.77 (226.32)	3.94 (0.24)	0	0	1 (5.6%)	17 (94.4%)	2.72 (1.07)	0.94 (0.64)
	27	20 (4.1%)	475.4 (296.3) [0]	89 - 1035	6.15 (1.42)	4 - 9	827.03 (360.19)	275.67 - 1799.68	887.56 (337.67)	3.90 (0.31)	0	0	2 (10%)	18 (90%)	1.30 (0.66)	0.65 (0.59)
	ALL	201 (41.2%)	405.30 (242.79) [9]	42 - 1553	5.54 (1.45)	2 - 10	799.22 (411.21)	62.35 - 1912.00	860.62 (420.93)	3.53 (0.62)	0	14 (7%)	67 (33.3%)	120 (59.7%)	1.33 (1.17)	0.63 (0.55)
CDS – Mostly, only pre-school ages	3	20 (4.1%)	221.1 (101.6) [0]	81 – 421	6.40 (3.22)	2 - 9	904.16 (555.90)	129.83 – 2059.25	997.36 (668.34)	3.25 (0.97)	0	7 (35%)	1 (5%)	12 (60%)	0.65 (0.87)	0.70 (0.73)
	15	20 (4.1%)	345.5 (131.1) [0]	161 - 662	5.25 (1.59)	3 - 8	1050.60 (193.22)	720.60 – 1519.42	1125.64 (184.80)	3.80 (0.41)	0	0	4 (20%)	16 (80%)	1.60 (0.60)	0.85 (0.81)
	18	21 (4.3%)	381.7 (174.2) [0]	174 - 860	7.57 (0.98)	6 - 9	825.14 (147.48)	679.00 – 1217.00	949.96 (151.98)	3.86 (0.36)	0	0	3 (14.3%)	18 (85.7%)	1.62 (1.07)	2.20 (1.03)

	19	20 (4.1%)	61.0 (n/a) [19]	61 – 61	1.90 (0.31)	1 - 2	614.43 (186.23)	225.50 – 1130.33	732.17 (211.75)	1.10 (0.31)	18 (90%)	2 (10%)	0	0	2.40 (0.50)	1.50 (0.69)
	ALL	81 (16.6%)	313.02 (155.88)[19]	61 - 860	5.31 (2.80)	1 - 9	848.29 (347.51)	129.83 - 2059.25	951.27 (388.27)	3.01 (1.26)	18 (22.2%)	9 (11.1%)	8 (9.9%)	46 (56.8%)	1.57 (1.00)	1.32 (1.01)
All CDS		394 (80.7%)	384.40 (234.61)	41 - 1553	5.20 (1.94)	1 - 10	804.38 (432.92)	62.35 (3366.00)	875.65 (450.35)	3.28 (0.92)	40 (8.2%)	96 (19.7%)	122 (25.0%)	230 (47.1%)	1.38 (1.18)	0.73 (0.74)
Grand total		**27 sites N=488**	**374.53 (235.03)[54]**	**41 - 1553**	**4.93 (2.02)**	**1 - 10**	**846.00 (536.31)**	**0.00 – 4087.05**	**922.41 (547.31)**	**3.11 (0.99)**	**40 (8.2%)**	**96 (19.7%)**	**122 (25.0%)**	**230 (47.1%)**	**1.51 (1.17)**	**0.64 (0.71)**

#Number of professional encounters scored by adding the exposure to paediatrician, psychologist, SALT and nurse/HV/CNN at each of the four stages: (filtering, referral, two further assessments, maximum possible=16

Hours of professional time – Summary table

	CDS N=394 Missing 0	CAHMS N=77 Missing 0	Tertiary N=17 Missing 0	Mann Whitney U test
Mean	10.50	13.26	26.77	CDS vs CAHMS p<0.001 CDS vs Tertiary p<0.001 CAHMS vs Tertiary p<0.001
Median	9.21	12.75	27.30	
Std. Deviation	5.75	5.58	15.99	
Minimum	0.85	1.50	0.00	
Maximum	35.72	30.50	60.90	
Percentiles25	6.83	9.62	14.46	
Percentiles 75	13.50	16.25	37.97	

Costs summary table and notes

	CDS N=394 Missing 0	CAHMS N=77 Missing 0	Tertiary N=17 Missing 0	Mann Whitney U test
Mean	804.38	790.81	2060.62	CDS vs CAHMS p=0.906 CDS vs Tertiary p<0.001 CAHMS vs Tertiary p<0.001
Median	723.25	726.00	1881.10	
Std. Deviation	432.92	381.19	1355.02	
Minimum	62.35	97.50	.00	
Maximum	3366.00	2024.00	4087.05	
Percentiles25	518.19	547.00	926.57	
Percentiles 75	1040.27	1010.75	3381.47	

Unit costs were drawn from Curtis L and Burns A. Unit costs of Health and Social Care 2020, University of Kent, PSSRU (16), as follows:

- Cost per working hour of medical consultant £119/ hour (Section 14, hospital based doctor)
- Scientific and Professional Staff, section 9, Community based staff – £69 per hour for Psychologist and Other, grade 8a; and £58 per working hour for SALT, grade 7
- Section 10, nurses, Band 6, £49 per working hour

Total cost of appointments for each child for each professional was the product of the unit costs and time spent with that professional. Costs by professional were summed for each child.

Note:

- All costs are fully absorbed, i.e. include staff oncosts, and overheads (management, administrative, estates, non staff). For this reason, the separate recording of administrative staff is excluded from the calculation. In addition, it was thought that administrative input was inconsistently reported across sites.
- DNAs were excluded because they were inconsistently reported.
- Costs based on 1 hour per consultation (on advice of clinical team members).

Supplementary Table 3: Modelling of days to diagnosis and total costs to diagnosis (excluding follow up)

```
*** TAKE LOGS OF DEPENDENT VARIABLES.
if (DaystoDiag>0) LogDaystoDiag=ln(DaystoDiag).
graph /histogram(normal) logdaystodiag.
```

Graph

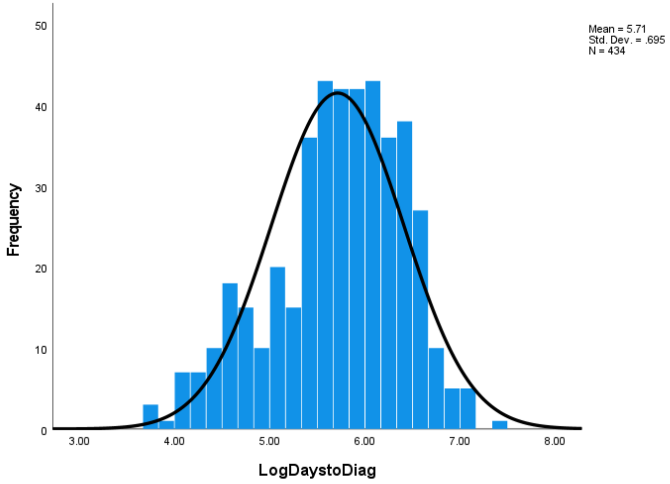

**Final Model** R^2 = .021	Unstandardized Coefficients		Standardized Coefficients			95.0% Confidence Interval for B	
	B	Std. Error	Beta	t	Sig	Lower Bound	Upper Bound
(Constant)	5.665	.035		159.877	.000	5.595	5.735
FinalDiag6	.316	.104	.146	3.052	.002	.113	.520

So Loge(Days to Diagnosis) = 5.665 + 0.226*FinalDiag6 (1=Yes,0=No) i.e. Predicted Days to Diagnosis = exp(5.665 + 0.316*FinalDiag6 (1=Yes,0=No))

EXAMPLES
ANY age child with Final Diagnosis = 1 or 2 or 3 or 4 or 5: Predicted assessment days to diagnosis = 288.6 days
ANY age child with Final Diagnosis = 6 : Predicted assessment days to diagnosis = 395.8 days

Age in months was not significant

```
if (TOTALCOSTexcFU>0)  LogTOTALCOSTexcFU=ln(TOTALCOSTexcFU).
graph /histogram(normal) LogTOTALCOSTexcFU.
```

Graph

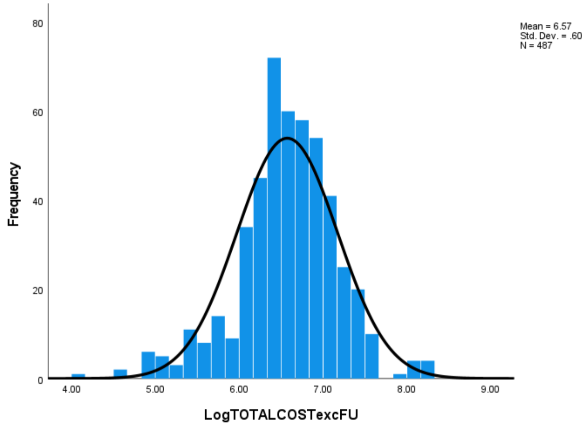

**Final Model** R^2 = .125	Unstandardized Coefficients		Standardized Coefficients			95.0% Confidence Interval for B	
	B	Std. Error	Beta	t	Sig	Lower Bound	Upper Bound
(Constant)	6.217	.054		115.771	.000	6.111	6.322
No. of assessment days	.001	.000	.277	6.028	<.001	.000	.001
FinalDiag4	.164	.060	.136	2.729	.007	.046	.282
FinalDiag5	.285	.068	.205	4.169	<.001	.151	.420
FinalDiag6	.223	.081	.134	2.746	.006	.063	.383

So Loge (Total Cost excluding Follow-Up) =
6.217 +0.001*(No. of days from Filtering to final assessment session) +0.164*FinalDiag4(1=Yes,0=No) +0.285*FinalDiag5(1=Yes,0=No) +0.223*FinalDiag6(1=Yes,0=No)
So Predicted Total Cost excluding Follow-Up = exp(above formula)

EXAMPLES
Days to diagnosis=365 and Final Diagnosis=1 or 2 or 3: Predicted Total Cost excluding Follow-Up = £721.98
Days to diagnosis=365 and Final Diagnosis =4: Predicted Total Cost excluding Follow-Up = £850.65
Days to diagnosis=365 and Final Diagnosis =5: Predicted Total Cost excluding Follow-Up = £960.06
Days to diagnosis=365 and Final Diagnosis =6: Predicted Total Cost excluding Follow-Up = £902.35
Days to diagnosis=183 and Final Diagnosis =1 or 2 or 3: Predicted Total Cost excluding Follow-Up = £601.85
Days to diagnosis=183 and Final Diagnosis =6: Predicted Total Cost excluding Follow-Up = £752.20
